# The impact of fatty acids biosynthesis on the risk of cardiovascular diseases in Europeans and East Asians: a Mendelian randomization study

**DOI:** 10.1093/hmg/ddac153

**Published:** 2022-07-07

**Authors:** Maria-Carolina Borges, Phillip Haycock, Jie Zheng, Gibran Hemani, Laurence J Howe, A Floriaan Schmidt, James R Staley, R Thomas Lumbers, Albert Henry, Rozenn N Lemaitre, Tom R Gaunt, Michael V Holmes, George Davey Smith, Aroon D Hingorani, Deborah A Lawlor

**Affiliations:** MRC Integrative Epidemiology Unit, University of Bristol, Bristol BS8 2BN, UK; Population Health Sciences, Bristol Medical School, University of Bristol, Bristol BS8 2PN, UK; MRC Integrative Epidemiology Unit, University of Bristol, Bristol BS8 2BN, UK; Population Health Sciences, Bristol Medical School, University of Bristol, Bristol BS8 2PN, UK; MRC Integrative Epidemiology Unit, University of Bristol, Bristol BS8 2BN, UK; Population Health Sciences, Bristol Medical School, University of Bristol, Bristol BS8 2PN, UK; MRC Integrative Epidemiology Unit, University of Bristol, Bristol BS8 2BN, UK; Population Health Sciences, Bristol Medical School, University of Bristol, Bristol BS8 2PN, UK; MRC Integrative Epidemiology Unit, University of Bristol, Bristol BS8 2BN, UK; Population Health Sciences, Bristol Medical School, University of Bristol, Bristol BS8 2PN, UK; Faculty of Population Health Sciences, Institute of Cardiovascular Science, University College London, London WC1E 6DD, UK; Department of Cardiology, Division Heart and Lungs, UMC Utrecht, Utrecht 3584 CX, The Netherlands; MRC Integrative Epidemiology Unit, University of Bristol, Bristol BS8 2BN, UK; Population Health Sciences, Bristol Medical School, University of Bristol, Bristol BS8 2PN, UK; Institute of Health Informatics, University College London, London NW1 2DA, UK; Health Data Research UK London, University College London NW1 2DA, UK; UCL British Heart Foundation Research Accelerator, London NW1 2DA, UK; Faculty of Population Health Sciences, Institute of Cardiovascular Science, University College London, London WC1E 6DD, UK; Institute of Health Informatics, University College London, London NW1 2DA, UK; UCL British Heart Foundation Research Accelerator, London NW1 2DA, UK; Cardiovascular Health Research Unit, Department of Medicine, University of Washington, Seattle, WA WA 98101, USA; MRC Integrative Epidemiology Unit, University of Bristol, Bristol BS8 2BN, UK; Population Health Sciences, Bristol Medical School, University of Bristol, Bristol BS8 2PN, UK; Medical Research Council Population Health Research Unit, University of Oxford, Oxford OX3 7LF, UK; Clinical Trial Service and Epidemiological Studies Unit, Nuffield Department of Population Health, University of Oxford, Oxford OX3 7LF, UK; MRC Integrative Epidemiology Unit, University of Bristol, Bristol BS8 2BN, UK; Population Health Sciences, Bristol Medical School, University of Bristol, Bristol BS8 2PN, UK; Faculty of Population Health Sciences, Institute of Cardiovascular Science, University College London, London WC1E 6DD, UK; Health Data Research UK London, University College London NW1 2DA, UK; UCL British Heart Foundation Research Accelerator, London NW1 2DA, UK; MRC Integrative Epidemiology Unit, University of Bristol, Bristol BS8 2BN, UK; Population Health Sciences, Bristol Medical School, University of Bristol, Bristol BS8 2PN, UK; NIHR Bristol Biomedical Research Centre, Bristol BS8 2BN, UK

## Abstract

Despite early interest, the evidence linking fatty acids to cardiovascular diseases (CVDs) remains controversial. We used Mendelian randomization to explore the involvement of polyunsaturated (PUFA) and monounsaturated (MUFA) fatty acids biosynthesis in the etiology of several CVD endpoints in up to 1 153 768 European (maximum 123 668 cases) and 212 453 East Asian (maximum 29 319 cases) ancestry individuals. As instruments, we selected single nucleotide polymorphisms mapping to genes with well-known roles in PUFA (i.e. *FADS1/2* and *ELOVL2*) and MUFA (i.e. *SCD*) biosynthesis. Our findings suggest that higher PUFA biosynthesis rate (proxied by rs174576 near *FADS1/2*) is related to higher odds of multiple CVDs, particularly ischemic stroke, peripheral artery disease and venous thromboembolism, whereas higher MUFA biosynthesis rate (proxied by rs603424 near *SCD*) is related to lower odds of coronary artery disease among Europeans. Results were unclear for East Asians as most effect estimates were imprecise. By triangulating multiple approaches (i.e. uni-/multi-variable Mendelian randomization, a phenome-wide scan, genetic colocalization and within-sibling analyses), our results are compatible with higher low-density lipoprotein (LDL) cholesterol (and possibly glucose) being a downstream effect of higher PUFA biosynthesis rate. Our findings indicate that PUFA and MUFA biosynthesis are involved in the etiology of CVDs and suggest LDL cholesterol as a potential mediating trait between PUFA biosynthesis and CVDs risk.

## Introduction

Fatty acids constitute the main components of dietary fats and are required in human nutrition as a source of energy and for metabolic and structural activities (1). They are capable of influencing a wide range of cell signaling pathways and have been implicated in the regulation of several processes involved in the etiology of cardiovascular diseases (CVDs), including lipid metabolism (2–4), glucose homeostasis (5,6), blood pressure (7–9), inflammatory response (10–12) and endothelial function (9,13). Fatty acids are commonly subdivided into broad classes according to the degree of unsaturation (i.e. number of carbon–carbon double bonds) into saturated (SFA), monounsaturated (MUFA) and polyunsaturated (PUFA) fatty acids, the latter being classified as omega-3 or omega-6 PUFA depending on the position of the first double bond from the terminal methyl group.

**Figure 1:**
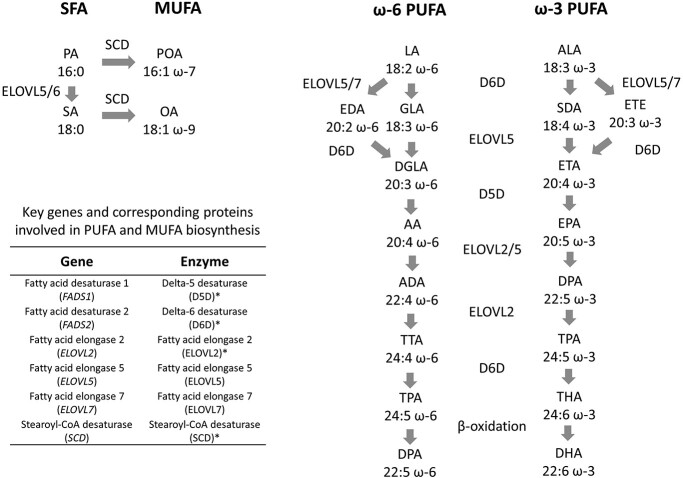
Overview of desaturation and elongation reactions involved in the conversion of MUFA from SFA and of longer-chain omega-3 and omega-6 PUFA from their shorter chain precursors. ^*^D5D, D6D, ELOVL2 and SCD activities were explored in the current study. Abbreviations: *Saturated fatty acids (SFA):* PA: palmitic acid; SA: stearic acid. *Monounsaturated fatty acids (MUFA):* POA: palmitoleic acid; OA: oleic acid. *ω-6 polyunsaturated fatty acids (PUFA):* LA: linoleic acid; GLA: ϒ-linolenic acid; EDA: eicosadienoic acid; DGLA: dihomo-ϒ-linolenic acid; AA: arachidonic acid; ADA: adrenic acid; TTA: tetracosatetraenoic acid; TPA: tetracosapentaenoic acid; DPA: docosapentaenoic acid. *ω-3 polyunsaturated fatty acids (PUFA):* ALA: α-linolenic acid; SDA: stearidonic acid; ETE: eicosatrienoic acid; ETA: eicosatetraenoic acid; EPA: eicosapentaenoic acid; DPA: docosapentaenoic acid; TPA: tetracosapentaenoic acid; THA: tetracosahexaenoic acid; DHA: docosahexaenoic acid.

Some fatty acids can be synthesized endogenously by fatty acid synthase or can be taken up by diet and further elongated and desaturated into longer chain fatty acids by fatty acid elongases and desaturases, respectively (14). Genome-wide association studies (GWAS) have reported that circulating fatty acids are strongly influenced by genetic variants near genes coding fatty acid elongases and desaturases: fatty acid desaturase 1 (*FADS1—*ENSG00000149485), fatty acid desaturase 2 (*FADS2—*ENSG00000134824), elongase 2 (*ELOVL2*—ENSG00000197977) and stearoyl-CoA desaturase (*SCD*—ENSG00000099194) (15–21). The chemical reactions and pathways catalyzed by the enzymes encoded by *FADS1*, *FADS2*, *ELOVL2* and *SCD* are summarized in Figure 1.

Mendelian randomization uses genetic variants associated with putative risk factors as instruments to assess their involvement in disease etiology (22–24). The use of human genetics to explore the effect of modifiable risk factors on cardiometabolic diseases, such as in Mendelian randomization, has proven valuable to (de)prioritize targets for intervention and to assess potential target-mediated adverse effects reducing late-stage failures in RCTs owing to lack of efficacy or from target-mediated adverse reactions (25).

Genetic variants affecting the expression or activity of genes encoding for fatty acid elongases and desaturases (e.g. *FADS1/2*, *ELOVL2* and *SCD*) can be used as causal anchors in Mendelian randomization studies investigating the involvement of fatty acids in the development of CVDs. Most previous Mendelian randomization studies investigating the role of fatty acids on the risk of CVDs have solely or heavily relied on genetic variants within the locus harboring *FADS1* and *FADS2*, which are involved in PUFA synthesis by encoding the enzymes delta-5 desaturase (D5D) and delta-6 desaturase (D6D), respectively. Overall, these studies have reported that shorter chain PUFA [e.g. α-linolenic acid (ALA) and linoleic acid (LA)] and longer chain PUFA [e.g. arachidonic acid (AA)] are associated with lower and higher risk of CVDs, respectively (26–32).

These studies potentially strengthen the evidence on the involvement of fatty acids in the development of CVDs, given the well-established link of D5D/D6D with PUFA biosynthesis. However, such studies suffer from a critical limitation, given *FADS1/2* variants are not reliable instruments for individual fatty acids. First, *FADS1/2* variants will affect multiple fatty acids on the same pathway and, in some cases, on different pathways with reactions catalyzed by D5D/D6D (Fig. 1). Second, these studies have not extensively explored whether the association of *FADS1/2* variants with CVDs risk could be explained by biological pathways independent of fatty acids (e.g. if variants simultaneously influence the expression of other genes in the region that affect CVDs) or owing to confounding by linkage disequilib-rium (LD), population stratification or other familial mechanisms.

The aim of this study was to use Mendelian randomization to explore the effect of fatty acids biosynthesis on a wide range of CVD end-points in up to 1 153 768 European and 212 453 East Asian ancestry individuals. We extend work in previous studies by using genetic variants regulating multiple rate-limiting enzymes in fatty acids biosynthesis (i.e. D5D/D6D, ELOVL2 and SCD), comparing findings between Europeans and East Asians and extensively exploring the key scenarios that could lead to spurious findings in this and previous Mendelian randomization studies.

## Results

### Genetic instruments indexing fatty acids biosynthesis

We selected genetic variants mapping to genes with a well-known role in fatty acids biosynthesis (i.e. *FADS1/2*, *ELOVL2* and *SCD*). To circumvent limitations from previous studies, we used genetic variants to instrument for enzyme activity in a given fatty acids biosynthesis pathway (rather than for individual fatty acids) by deriving the ratio between fatty acids that are the product and the substrate of a reaction catalyzed by the corresponding enzyme. This allows harnessing the advantages of *cis*-acting variants in the vicinity of genes coding for key enzymes in fatty acids biosynthesis pathways and can provide more credible evidence on the likely therapeutic effect of targeting such proteins in preventing CVDs (33).

In individuals of European ancestry, the selected genetic variants were rs174546 (*FADS1*, chr11q13.3), rs174576 (*FADS2*), rs3734398 (*ELOVL2*) and rs603424 (*SCD*), which explained a proportion of the variance in the corresponding marker of enzyme activity of 32.6% (*F* = 4174) for AA:DGLA [i.e. ratio between AA and dihomo-γ-linolenic acid (DGLA)], 6.3% (*F* = 580) for GLA:LA [i.e. the ratio between γ-linolenic acid (GLA) and LA], 2.4% (*F* = 218) for DHA:n-3 DPA [i.e. the ratio between docosahexaenoic acid (DHA) and omega-3 docosapentaenoic acid (DPA)] and 1.1% (*F* = 100) for POA:PA [i.e. the ratio between palmitoleic acid (POA) and palmitic acid (PA)], respectively (Supplementary Material, Table S1). The *FADS1/2* single nucleotide polymorphisms (SNPs) (i.e. rs174546 and rs174576) were in strong LD (*R*^2^ = 0.931000 Genomes European population), and, therefore, only the SNP more strongly associated with the corresponding marker of enzyme activity was used in subsequent analyses (i.e. rs174546).

In individuals of East Asian ancestry, the top variant in the *FADS1/2* locus was palindromic and, therefore, was replaced by rs174546 (i.e. LD R^2^ = 0.93 in 1000G East Asian population), which explained 8.4% (*F* = 125) of the variance in DGLA:LA, a marker of D6D activity (Supplementary Material, Table S1). No genetic variants were associated with markers of D5D, ELOVL2 or SCD activity and, therefore, rs174546 was the only genetic variant eligible for further analyses in East Asians.

### Impact of genetic instruments on circulating fatty acids

Overall, the effect of genetic variants on the fatty acids pool was replicable across independent samples and between Europeans and East Asians. As expected, the genetic variants impact on the fatty acids pool was consistent with their predicted function on fatty acids biosynthesis. The *FADS1/2* SNP (rs174546) was associated with a lower concentration of shorter chain omega-3 (e.g. ALA) and omega-6 (e.g. LA) fatty acids and higher concentration of longer chain omega-3 (e.g. DHA) and omega-6 (e.g. AA) fatty acids. The *ELOVL2* SNP (rs3734398) was mostly associated with higher concentration of DHA and lower concentration of eicosapentaenoic acid (EPA) and n-3 DPA, whereas the *SCD* SNP (rs603424) was related to lower SFA, particularly PA, and higher MUFA, particularly POA (Supplementary Material, Figs S1 and S2).

### Relation between fatty acids biosynthesis and risk of CVDs

**Figure 2:**
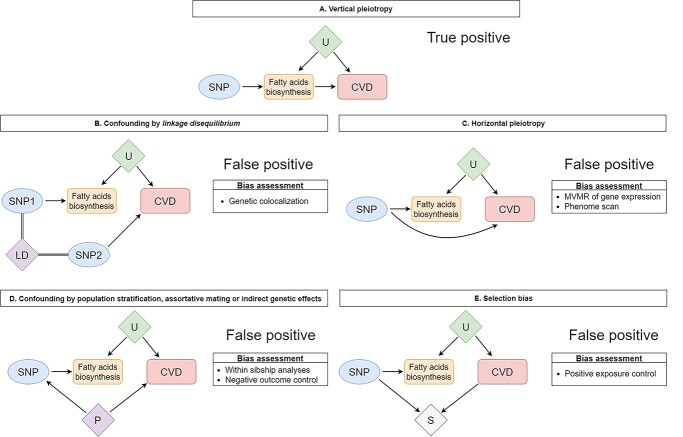
Schematic representation of scenarios leading to true (**A**) and spurious (**B**–**E**) findings in Mendelian randomization analyses on the effect of fatty acids biosynthesis and CVD risk. Image (A) represents vertical pleiotropy in which the effect of genetic instruments on CVD is mediated by fatty acids biosynthesis. Images (B–E) represent alternative mechanisms that could bias Mendelian randomization findings: (B) confounding by LD in which the selected genetic variant is in LD with another genetic variant influencing CVD independently; (C) horizontal pleiotropy in which the genetic variant influences fatty acids biosynthesis and CVD via two different biological pathways; (D) confounding by population stratification, assortative mating or indirect genetic effects, in which different phenomena can introduce spurious association between genetic variant and CVD in samples of unrelated individuals and (E) selection bias in which selection into the study creates a spurious association between the genetic variant and CVD owing to collider stratification bias. U: unobserved confounders; P: population phenomena (i.e. population stratification, assortative mating or indirect genetic effects); S: selection; SNP: single nucleotide polymorphism; CVD: cardiovascular diseases; LD: linkage disequilibrium.

We used two-sample Mendelian randomization to probe the lifelong effect of fatty acids biosynthesis on CVDs risk and risk factors in individuals of European and East Asian ancestry. Confounding by LD is a key source of bias in Mendelian randomization analyses using one or few independent genetic variants (Fig. 2) and occurs when the selected genetic instrument is correlated (i.e. in LD) with another genetic variant influencing the outcome independently. Therefore, we used genetic colocalization to tease apart whether results from Mendelian randomization analyses were compatible with a shared variant between enzyme activity markers and CVD outcomes or with confounding by LD.

#### *FADS* locus: D5D activity in Europeans

**Figure 3:**
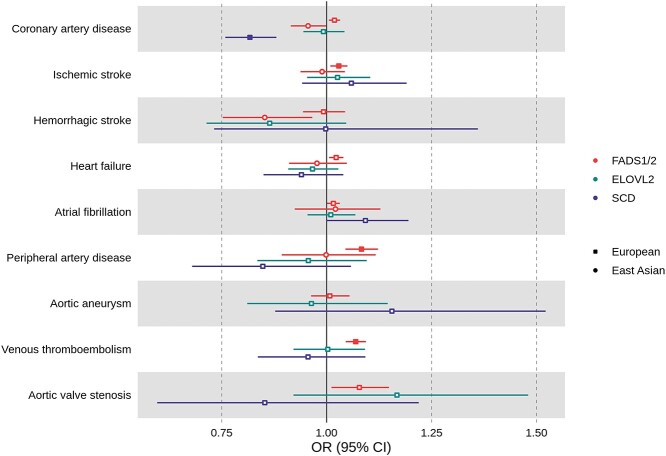
Mendelian randomization results for the risk of CVDs related to increasing activity of enzymes coded by *FADS1/2* (D5D/D6D), *ELOVL2* (ELOVL2) and *SCD* (SCD) among individuals of European and East Asian ancestries. Results are expressed as odds ratio of CVDs per standard unit increase in the marker of enzyme activity for *FADS1/2* (i.e. AA:DGLA ratio in Europeans and DGLA:LA ratio in East Asians), *ELOVL2* (i.e. DHA:DPA ratio in Europeans) and *SCD* (i.e. POA:PA ratio in Europeans) loci. For individuals of European ancestry, SNP-CVDs association data were metanalyzed across multiple genetic association consortia, UK Biobank and FinnGen. For individuals of East Asian ancestry, SNP-CVDs association data were extracted from BioBank Japan. Full symbols indicate associations at *P*-value lower than the *P*-value threshold accounting for multiple testing (*P* < 0.00556). AA: arachidonic acid; DGLA: dihomo-ϒ-linolenic acid; DHA: docosahexaenoic acid; DPA: docosapentaenoic acid; LA: linoleic acid; PA: palmitic acid; POA: palmitoleic acid; SNP: single nucleotide polymorphism; *FADS1/2:* fatty acid desaturases 1 and 2; ELOVL2: elongase 2; SCD: stearoyl-CoA desaturase.

**Figure 4:**
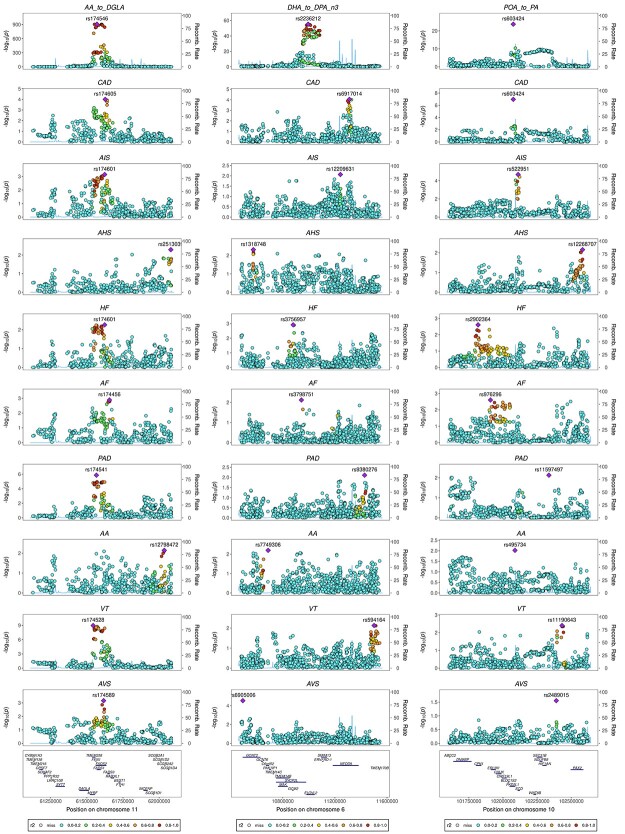
Genetic association plots for fatty acids enzyme activity (proxied by AA:DGLA, DHA:DPA and POA:PA ratios) and CVDs risk among individuals of European ancestry. Results for each trait are expressed as log_10_  *P*-values for the *FADS*, *ELOVL2* and *SCD* locus (columns 1, 2 and 3, respectively). AA: arachidonic acid; DGLA: dihomo-γ-linoleic acid; DHA: docosahexaenoic acid; DPA: docosapentaenoic acid; LA: linoleic acid; PA: palmitic acid; POA: palmitoleic acid; *FADS:* fatty acids desaturase; *ELOVL2:* elongase 2; *SCD:* stearoyl-CoA desaturase; CAD: coronary artery disease; AIS: any ischemic stroke; AHS: any haemorrhagic stroke; HF: heart failure; AF: atrial fibrillation; PAD: peripheral artery disease; AA: aortic aneurysm; VT: venous thromboembolism; AVS: aortic valve stenosis.

Mendelian randomization analyses in European ancestry individuals suggested that higher D5D activity [proxied by increase in AA:DGLA in standard deviation (SD) units] was related to higher odds of multiple CVDs, such as coronary artery disease [CAD; OR = 1.02; 95% confidence interval (CI): 1.01, 1.03; *P*-value = 0.006], ischemic stroke (OR = 1.03; 95% CI: 1.01, 1.05; *P*-value = 0.004), heart failure (HF; OR = 1.02; 95% CI: 1.01, 1.04; *P*-value = 0.008), atrial fibrillation (AF; OR = 1.02; 95% CI: 1.00, 1.03; *P*-value = 0.04), peripheral artery disease (PAD; OR = 1.08; 95% CI: 1.04, 1.12; *P*-value = $1\times{10}^{-5}$), venous thromboembolism (VT; OR = 1.07; 95% CI: 1.05, 1.09; *P*-value = $5\times{10}^{-9}$) and aortic valve stenosis (AVS; OR = 1.08; 95% CI: 1.01, 1.15; *P*-value = 0.02). Only results for ischemic stroke, PAD and VT passed our threshold for multiple testing correction (Fig. 3). Overall, results were consistent across studies except for CAD, for which the estimated effect was attenuated in UK Biobank compared with other studies, and for aortic aneurysm (AA), for which the estimated effect was in different directions between UK Biobank and other studies (Supplementary Material, Fig. S3). There was strong evidence of genetic colocalization between D5D activity and risk for VT as evidenced by a posterior probability of association (PPA) of 85% for a shared variant. For other CVD outcomes, PPA was 0–27% for a shared variant accompanied by PPA of 60–100% for the variant being associated with D5D activity only, which could be a result of limited statistical power (Table 1 and Fig. 4).

**Table 1:** Genetic colocalization results for enzyme activity and CVDs risk among European ancestry individuals

Locus	Exposure	Outcome	PPA H1: exposure only	PPA H2: outcome only	PPA H3: distinct variants	PPA H4: shared variant	PPA H5: none
*FADS1*	AA:DGLA	AA	1	0	0	0	0
*FADS1*	AA:DGLA	AF	1	0	0	0	0
*FADS1*	AA:DGLA	Hemorrhagic stroke	1	0	0	0	0
*FADS1*	AA:DGLA	Ischemic stroke	0.99	0	0	0	0
*FADS1*	AA:DGLA	AVS	1	0	0	0	0
*FADS1*	AA:DGLA	CAD	0.99	0	0	0	0
*FADS1*	AA:DGLA	HF	1	0	0	0	0
*FADS1*	AA:DGLA	PAD	0.61	0	0.12	0.27	0
*FADS1*	AA:DGLA	VT	0	0	0.15	0.85	0
*ELOVL2*	DHA:DPA_n3	AA	1	0	0	0	0
*ELOVL2*	DHA:DPA_n3	AF	1	0	0	0	0
*ELOVL2*	DHA:DPA_n3	Hemorrhagic stroke	1	0	0	0	0
*ELOVL2*	DHA:DPA_n3	Ischemic stroke	1	0	0	0	0
*ELOVL2*	DHA:DPA_n3	AVS	0.99	0	0.01	0	0
*ELOVL2*	DHA:DPA_n3	CAD	0.99	0	0	0	0
*ELOVL2*	DHA:DPA_n3	HF	1	0	0	0	0
*ELOVL2*	DHA:DPA_n3	PAD	1	0	0	0	0
*ELOVL2*	DHA:DPA_n3	VT	1	0	0	0	0
*SCD*	POA:PA	AA	1	0	0	0	0
*SCD*	POA:PA	AF	1	0	0	0	0
*SCD*	POA:PA	Hemorrhagic stroke	1	0	0	0	0
*SCD*	POA:PA	Ischemic stroke	0.99	0	0.01	0	0
*SCD*	POA:PA	AVS	1	0	0	0	0
*SCD*	POA:PA	CAD	0.01	0	0	0.99	0
*SCD*	POA:PA	HF	1	0	0	0	0
*SCD*	POA:PA	PAD	1	0	0	0	0
*SCD*	POA:PA	VT	1	0	0	0	0

Results are expressed as posterior probabilities of association (PPA) with fatty acid trait only (H1), cardiovascular trait only (H2), both traits owing to distinct causal variants (H3), both traits owing to a shared causal variant (H4), or no association (H5). *FADS1:* fatty acid desaturase 1; *ELOVL2:* elongase 2; *SCD:* stearoyl-CoA desaturase; AA:DGLA: arachidonic acid to dihomo-γ-linoleic acid ratio; DHA:DPA_n3: docosahexaenoic acid to omega-3 eicosapentaenoic acid ratio; POA:PA: palmitoleic acid to palmitic acid ratio.

**Figure 5:**
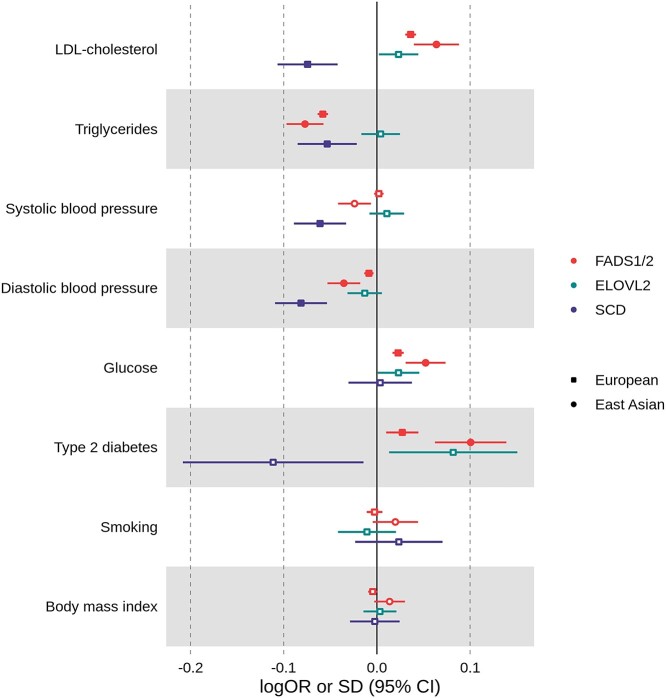
Mendelian randomization results for cardiovascular risk factors related to increasing activity of enzymes coded by *FADS1/2* (D5D/D6D), *ELOVL2* (ELOVL2) and *SCD* (SCD) among individuals of European and East Asian ancestries. Results are expressed as change in standard units (SD) or log odds ratio (logOR) of CVD risk factors per standard unit increase in the marker of enzyme activity for *FADS1/2* (i.e. AA:DGLA ratio in Europeans and DGLA:LA ratio in East Asians), *ELOVL2* (i.e. DHA:DPA ratio in Europeans) and *SCD* (i.e. POA:PA ratio in Europeans) loci. For individuals of European ancestry, data were extracted from UK Biobank or genetic association studies. For individuals of East Asian ancestry, data were extracted from BioBank Japan. Full symbols indicate associations at *P*-value lower than the *P*-value threshold accounting for multiple testing (*P* < 0.00625). Smoking is represent by pack years of smoking and number of cigarettes per day in European and East Asian ancestry individuals, respectively. AA: arachidonic acid; DGLA: dihomo-γ-linolenic acid; DHA: docosahexaenoic acid; DPA: docosapentaenoic acid; LA: linoleic acid; PA: palmitic acid; POA: palmitoleic acid; *FADS1/2:* fatty acid desaturases 1 and 2; *ELOVL2:* elongase 2; *SCD:* stearoyl-CoA desaturase; LDL-cholesterol: low-density lipoprotein-cholesterol.

**Figure 6:**
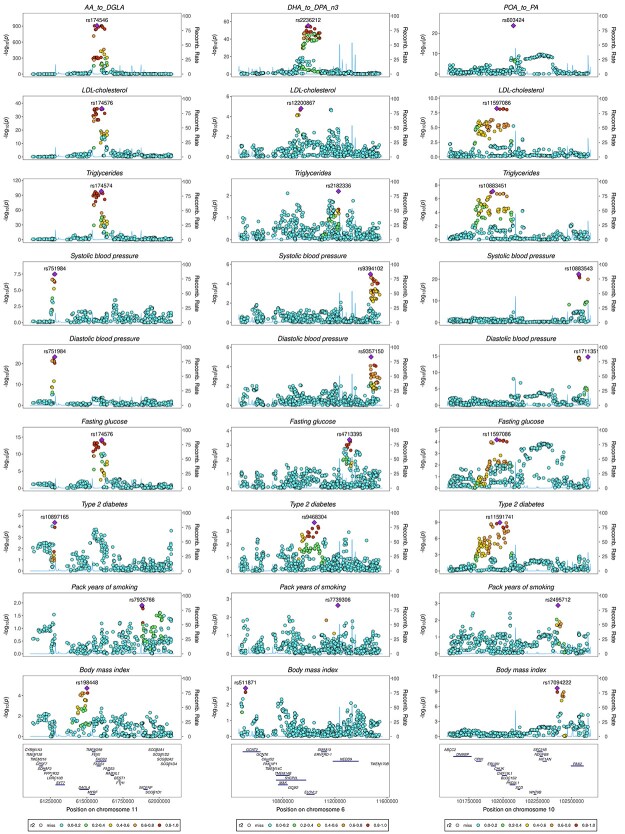
Genetic association plots for fatty acids enzyme activity (proxied by AA:DGLA, DHA:DPA and POA:PA ratio) and CVD risk factors among individuals of European ancestry. Results for each trait are expressed as log_10_  *P*-values for the *FADS, ELOVL2* and *SCD* locus (columns 1, 2 and 3, respectively). AA: arachidonic acid; DGLA: dihomo-γ-linoleic acid; DHA: docosahexaenoic acid; DPA: docosapentaenoic acid; LA: linoleic acid; PA: palmitic acid; POA: palmitoleic acid; *FADS:* fatty acids desaturase; *ELOVL2:* elongase 2; *SCD:* stearoyl-CoA desaturase; LDL: low-density lipoprotein.

Mendelian randomization analyses indicated that higher D5D activity was related to higher low-density lipoprotein (LDL) cholesterol, fasting glucose and type 2 diabetes risk, but lower triglycerides and diastolic blood pressure among individuals of European ancestry (Fig. 5). Genetic colocalization provided evidence for a shared variant between D5D activity and LDL cholesterol (PPA for shared variant = 87%) but not for systolic, diastolic blood pressure and triglycerides (PPA for distinct variants = 90–100%). Evidence was less conclusive for glucose (PPA for shared variant = 64%) (Table 2 and Fig. 6). In sensitivity analyses, support for a shared variant did not increase after conditioning the outcome genetic association data on the top genetic variant for the outcome (Supplementary Material, Table S2).

#### *FADS* locus: D6D activity in East Asians

In East Asian ancestry individuals, there was limited evidence from Mendelian randomization supporting a relationship between D6D activity (proxied by DGLA:LA in SD units) and the odds of cardiovascular endpoints. However, statistical power was substantially lower for analyses in East Asian individuals and, therefore, some findings could be compatible with higher D6D activity being related to higher odds of disease, such as for AF (OR = 1.02; 95% CI: 0.92, 1.13; *P*-value = 0.68), or to lower odds of diseases, such as for CAD (OR = 0.96; 95% CI: 0.91, 1.00; *P*-value = 0.04) and hemorrhagic stroke (OR = 0.85; 95% CI: 0.75, 0.97; *P*-value = 0.01) (Fig. 3). Higher D6D activity was related to higher LDL cholesterol, fasting glucose and type 2 diabetes risk, but lower triglycerides and diastolic blood pressure among individuals of East Asian ancestry (Fig. 5). We could not assess confounding by LD since genetic colocalization assumes that samples are drawn from independent populations of similar allele frequencies and LD pattern, which was not the case for East Asians in our analyses as genetic association data for fatty acids and CVD data were derived from Singaporean Chinese and Japanese individuals, respectively.

#### *ELOVL2* locus: ELOVL2 activity in Europeans

Mendelian randomization analyses did not support a relationship between higher ELOVL2 activity (proxied by increase in DHA:DPAn-3 in SD units) and cardiovascular endpoints. However, some results were imprecisely estimated and, therefore, we ca nnot rule out the presence of potentially important effects, particularly for hemorrhagic stroke (OR = 0.86; 95% CI: 0.71, 1.05; *P*-value = 0.14) and AVS (OR = 1.17; 95% CI: 0.92, 1.48; *P*-value = 0.20) (Fig. 3 and Supplementary Material, Fig. S3). Higher ELOVL2 activity was not related to cardiovascular risk factors at *P*-value < 0.00625 (Fig. 5).

#### *SCD* locus: SCD activity in Europeans

Higher SCD activity (proxied by increase in POA:PA in SD units) was related to lower odds of CAD (OR = 0.82; 95% CI: 0.76, 0.88; *P*-value = $1\times{10}^{-7}$) in Mendelian randomization analyses (Fig. 3), which was consistent across studies (Supplementary Material, Fig. S3). There was limited evidence supporting a relationship between higher SCD activity and other cardiovascular endpoints, although some of these results were imprecisely estimated and, therefore, we cannot rule out the presence of potentially important effects, particularly for PAD (OR = 0.85; 95% CI: 0.68, 1.06; *P*-value = 0.14), AA (OR = 1.16; 95% CI: 0.88, 1.52; *P*-value = 0.30) and AVS (OR = 0.85; 95% CI: 0.60, 1.22; *P*-value = 0.38) (Fig. 3 and Supplementary Material, Fig. S3). There was strong evidence that SCD activity colocalized with odds of CAD (PPA = 99% for a shared variant) (Table 1 and Fig. 4).

Higher SCD activity was related to lower LDL cholesterol, triglycerides, systolic and diastolic blood pressures (Fig. 5); however, colocalization analyses supported distinct genetic variants between POA:PA and these endpoints (PPA for distinct variants = 86–100%) (Table 2 and Fig. 6). In sensitivity analyses, support for a shared variant did not increase after conditioning the outcome genetic association data on the top genetic variant for the outcome in the genomic region except for DBP (PPA = 99%) (Supplementary Material, Table S2).

### Exploring bias in Mendelian randomization analyses

Apart from confounding by LD, other key sources of bias could invalidate inferences from this and previous Mendelian randomization studies, as detailed in Figure 2, including horizontal pleiotropy, where the genetic variant influences the outcome via a different biological pathway; confounding by population stratification, assortative mating or indirect genetic effects, which could create a spurious association between genetic variant and outcome in samples of unrelated individuals; and selection bias, where the genetic variant (or, more likely, its downstream traits) and the outcome affect selection into the sample resulting in a spurious association. We conducted extensive sensitivity analyses to explore the presence of such biases in our findings as detailed in the following text.

#### Horizontal pleiotropy

Horizontal pleiotropy is one of the main threats to the validity of Mendelian randomization studies since it is a widespread biological phenomenon and cannot be empirically verified. We used two approaches to explore the plausibility that our results are explained by horizontal pleiotropy: (i) a phenome-wide scan of the selected genetic variants using data from European and East Asian ancestry individuals and (ii) multivariable Mendelian randomization (MVMR) to estimate the direct effect of *FADS1*, *ELOVL2* and *SCD* expression on cardiovascular outcomes after accounting for the potential effect of other genes expressed in the corresponding genomic region using data from European ancestry individuals only (tissue-specific gene expression data was not available for East Asians).

**Figure 7:**
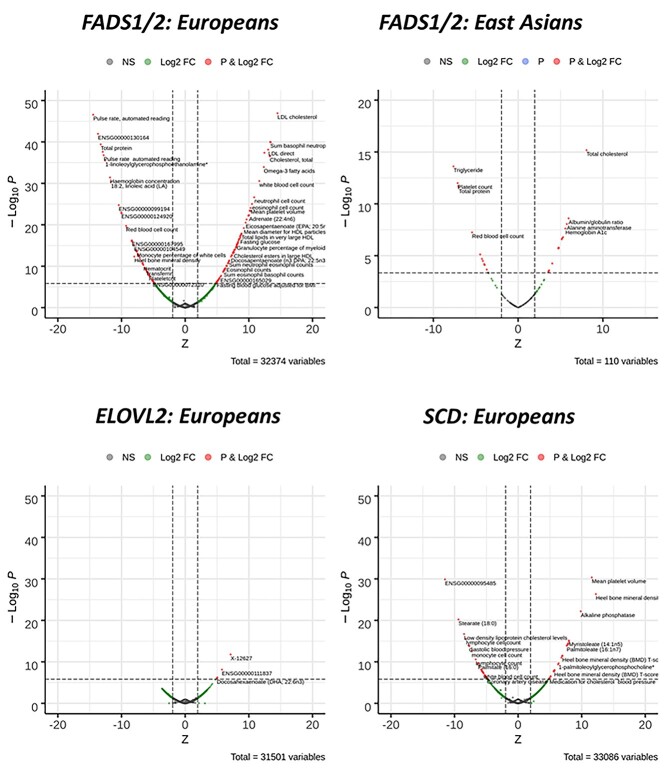
Phenome wide association scan of *FADS1/2* (rs174546), *ELOVL2* (rs3734398) and *SCD* (rs603424) genetic variants in European and East Asian ancestry individuals. Results are expressed as the *Z*-statistic for the variant-trait association for the allele increasing enzyme expression/activity. Red circles denote *P*-value < $1.5\times{10}^{-6}$ in Europeans and *P*-value < $4.5\times{10}^{-4}$ in East Asians. *FADS1/2:* fatty acid desaturases 1 and 2; *ELOVL2:* elongase 2; *SCD:* stearoyl-CoA desaturase.

In the phenome-wide scan, the *FADS1/2* variant (rs174546) was related not only to fatty acids but also to numerous non-fatty acid traits such as lipid, glycemic, blood cell traits, physical measures (e.g. pulse, heart rate and height), immune-related disorders (e.g. asthma, hypothyroidism, Crohn’s disease and inflammatory bowel disease) and several biomarkers (e.g. total bilirubin, insulin growth factor-1, cystatin C, alkaline phosphatase and urate) among individuals of European ancestry (Fig. 7 and Supplementary Material, Table S3). The pleiotropic associations of the *FADS1/2* variant (rs174546) were also seen in East Asians in relation to lipid, glycemic, blood cell traits (Fig. 7 and Supplementary Material, Table S4). The *ELOVL2* variant (rs3734398) was related to levels of an unknown metabolite X-12627 and DHA and the *SCD* variant (rs603424) was related to multiple SFA/MUFA as well as to bone mineral density and blood cell-related traits (Fig. 7 and Supplementary Material, Table S3).

**Table 2:** Genetic colocalization results for enzyme activity and cardiovascular risk factors among European ancestry individuals

Locus	Exposure	Outcome	PPA H1: Exposure only	PPA H2: Outcome only	PPA H3: distinct variants	PPA H4: shared variant	PPA H5: none
*FADS1*	AA:DGLA	Body mass index	0.98	0	0.02	0	0
*FADS1*	AA:DGLA	Diastolic blood pressure	0	0	1	0	0
*FADS1*	AA:DGLA	Glucose	0	0	0.36	0.64	0
*FADS1*	AA:DGLA	LDL cholesterol	0	0	0.13	0.87	0
*FADS1*	AA:DGLA	Systolic blood pressure	0.1	0	0.9	0	0
*FADS1*	AA:DGLA	Smoking	1	0	0	0	0
*FADS1*	AA:DGLA	Type 2 diabetes	0.95	0	0.03	0.02	0
*FADS1*	AA:DGLA	Triglycerides	0	0	1	0	0
*ELOVL2*	DHA:DPA_n3	Body mass index	0.61	0	0.39	0	0
*ELOVL2*	DHA:DPA_n3	Diastolic blood pressure	0.97	0	0.03	0	0
*ELOVL2*	DHA:DPA_n3	Glucose	0.99	0	0.01	0	0
*ELOVL2*	DHA:DPA_n3	LDL cholesterol	0.94	0	0.06	0	0
*ELOVL2*	DHA:DPA_n3	Systolic blood pressure	0.95	0	0.05	0	0
*ELOVL2*	DHA:DPA_n3	Smoking	1	0	0	0	0
*ELOVL2*	DHA:DPA_n3	Type 2 diabetes	0.99	0	0.01	0	0
*ELOVL2*	DHA:DPA_n3	Triglycerides	1	0	0	0	0
*SCD*	POA:PA	Body mass index	0	0	1	0	0
*SCD*	POA:PA	Diastolic blood pressure	0	0	1	0	0
*SCD*	POA:PA	Glucose	0.92	0	0.08	0	0
*SCD*	POA:PA	LDL cholesterol	0.01	0	0.95	0.03	0
*SCD*	POA:PA	Systolic blood pressure	0	0	1	0	0
*SCD*	POA:PA	Smoking	1	0	0	0	0
*SCD*	POA:PA	Type 2 diabetes	0	0	1	0	0
*SCD*	POA:PA	Triglycerides	0.14	0	0.86	0	0

Results are expressed as posterior probabilities of association (PPA) with fatty acid trait only (H1), cardiovascular trait only (H2), both traits owing to distinct causal variants (H3), both traits owing to a shared causal variant (H4), or no association (H5). *FADS1:* fatty acid desaturase 1; *ELOVL2:* elongase 2; *SCD:* stearoyl-CoA desaturase; AA:DGLA: arachidonic acid to dihomo-γ-linoleic acid ratio; DHA:DPA_n3: docosahexaenoic acid to omega-3 eicosapentaenoic acid ratio; POA:PA: palmitoleic acid to palmitic acid ratio.

Overall, the selected genetic instruments were strongly associated with the tissue expression of the target genes in the expected direction (Supplementary Material, Fig. S4), except for *FADS1* in whole blood, and genetic colocalization supported a shared variant between the enzyme activity proxies and the expression of the target gene in key tissues (Supplementary Material, Fig. S5 and Supplementary Material, Table S5). As an example, there was strong evidence that SCD activity (proxied by POA:PA) colocalized with *SCD* expression in adipose tissues (PPA = 100% for a shared variant), which are key tissues for *de novo* lipogenesis. The selected genetic variants were also associated with the expression of nearby non-target genes, which was particularly the case for the *FADS* variant (Supplementary Material, Fig. S4). The association of the genetic variants with the expression of non-target genes could bias our analyses if the proteins encoded by these genes directly influence CVDs. To explore that, we used MVMR, which supported a direct effect of the target genes (i.e. *FADS1*, *ELOVL2* and *SCD*) on CVDs and risk factors in individuals of European ancestry (Supplementary Material, Figs S6 and S7). The conditional *F* statistics for these analyses ranged from 18 to 433 and from 5 to 50 in unadjusted and adjusted models, respectively (Supplementary Material, Table S6).

#### Confounding by population stratification, assortative mating and indirect genetic effects

Mendelian randomization studies generally assume that genetic association estimates reflect the direct effect of a genetic variant on a phenotype, i.e. the downstream effect of inheriting an allele. However, there is growing evidence that genetic association estimates obtained from samples of unrelated individuals may also capture non-direct sources of association relating to population stratification, assortative mating and indirect genetic effects of parents (34). Of particular concern for this study, there is evidence that the *FADS1/2* locus was under important selection pressure in different populations and at different times, possibly as a response to dietary changes and the need for adequate supply of essential long-chain PUFA from precursors (35). Despite attempts to control for population stratification in genetic association data (e.g. by adjusting for genomic principal components), there could still be residual population structure as has recently been shown in UK Biobank (36). In addition, there is a possibility that indirect genetic effects of parents bias studies among unrelated individuals, given the literature suggesting that maternal genotype for *FADS1/2* variants might indirectly influence offspring outcomes via intrauterine effects and/or breastfeeding (37).

We used two approaches to explore the potential impact of confounding by population stratification, assortative mating and indirect genetic effects in our analyses: (i) testing for the association of the selected genetic instruments with two negative control outcomes (i.e. skin color and ease of skin tanning) and (ii) comparing within-sibling associations of the selected genetic variants with cardiovascular risk factors with estimates obtained from unrelated individuals.

The *FADS1/2* SNP (rs174546) was associated with both negative control outcomes among Europeans: skin color [mean change of 0.005 unit per C allele (*P*-value = $5\times{10}^{-5})$] and ease of skin tanning [mean change of −0.006 unit per C allele (*P*-value = 0.007$)$], while the *SCD* SNP was associated with ease of skin tanning [mean change of −0.006 unit increase per C allele (*P*-value = $0.037)$] (Supplementary Material, Table S7). Since these traits could not conceivably be affected by fatty acids biosynthesis, evidence for an association between genetic variants and these negative control outcomes is indicative of residual population stratification.

**Figure 8:**
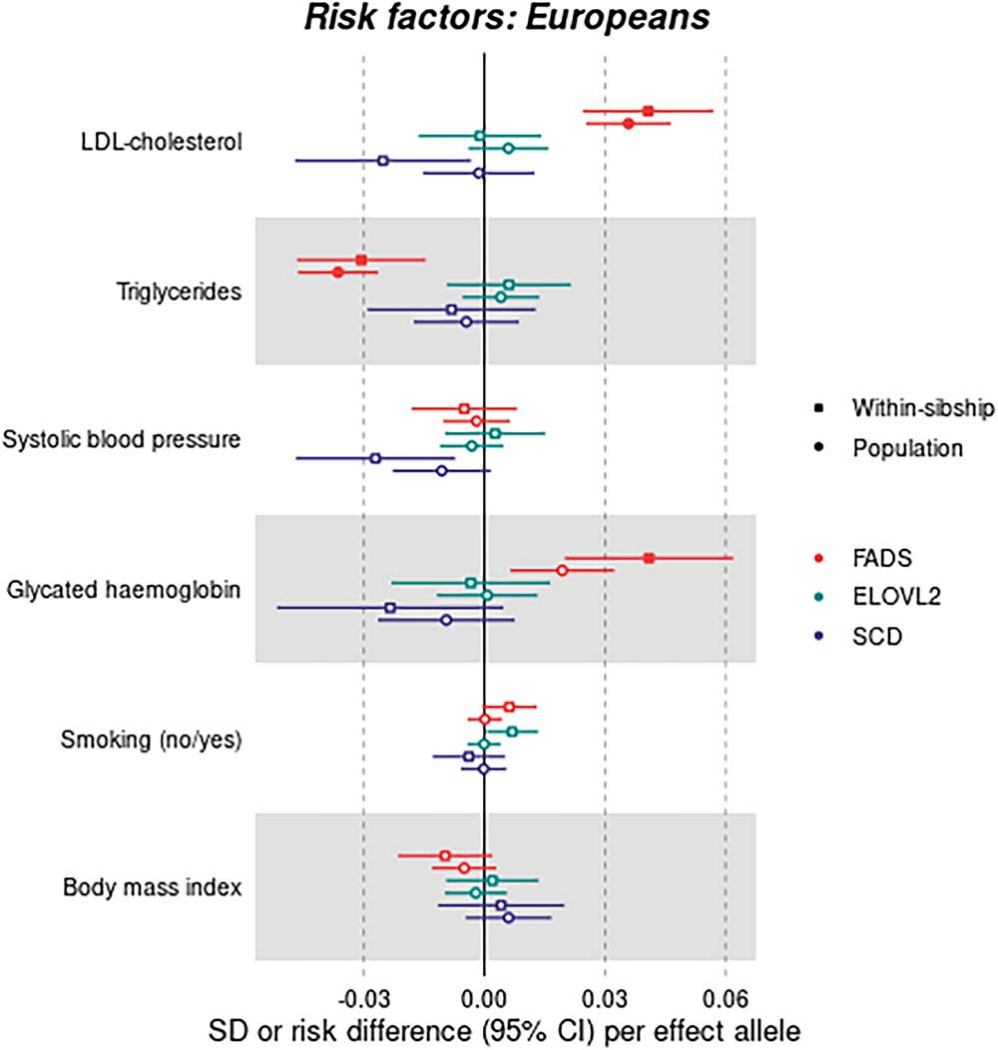
Association of *FADS1/2* (rs174546), *ELOVL2* (rs3734398) and *SCD* (rs603424) genetic variants with cardiovascular risk factors among unrelated individuals and within siblings of European ancestry. Results are expressed as change in SD units (or risk difference), and 95% CI, of cardiovascular risk factors per allele increasing enzyme activity. *FADS1/2:* fatty acid desaturases 1 and 2; *ELOVL2:* elongase 2; *SCD:* stearoyl-CoA desaturase.

We compared within-sibship associations of the selected genetic variants with cardiovascular risk factors with estimates obtained from unrelated individuals. Estimates were broadly consistent, indicating that our findings are unlikely to be substantially biased by population stratification, assortative mating or indirect genetic effects (Fig. 8). As an example, for the *FADS1/2* SNP (rs174546), each C allele was related to a mean LDL cholesterol increase of 0.041 (95% CI: 0.025; 0.057) and 0.036 (95% CI: 0.025; 0.046) SD units within-siblings and in unrelated individuals, respectively.

#### Selection bias

Several processes of sample selection, occurring from study design to data analyses, can result in selected samples not representative of their target populations, which may bias causal inference, including when using Mendelian randomization (38). We were particularly concerned about selection owing to ascertainment of CVD status as detailed in Materials and Methods. To explore whether these processes of sample selection could bias our findings, we adopted a positive exposure control approach in which we used Mendelian randomization to estimate the effect of well-established cardiovascular risk factors (i.e. LDL cholesterol, triglyc-erides, systolic, diastolic blood pressure, glucose, type 2 diabetes, smoking and body mass index) on the risk of CVDs. If the effects estimated in the positive control analyses were compatible with what expected and were comparable across data sources, such analyses would argue against selection being a major source of bias.

Overall, we observed the expected effect of well-established risk factors on the development of CVDs across studies (Supplementary Material, Fig. S8). Systolic, diastolic blood pressure and body mass index were related to higher odds of all CVD outcomes in studies of European ancestry individuals (i.e. UK Biobank, genetic association metanalyses and FinnGen) and higher odds of most cardiovascular outcomes (except for CAD and PAD) in Biobank Japan. Higher LDL cholesterol and triglycerides and liability to type 2 diabetes were related to higher odds of CAD and PAD across studies for both ancestries, while glucose and smoking were related to higher odds of PAD in Europeans and East Asians. There were a few instances where these risk factors were related to lower odds of disease, such as type 2 diabetes liability with hemorrhagic stroke (Biobank Japan) and LDL cholesterol with hemorrhagic stroke (UK Biobank and Biobank Japan).

## Discussion

### Main findings

In Europeans, our findings indicate that higher PUFA biosynthesis (proxied by *FADS1*/D5D activity) is related to higher risk of several CVDs (and risk factors), while higher MUFA biosynthesis (proxied by *SCD*/SCD activity) is related to lower risk of CAD. In addition, despite the strong LD in the *FADS1/2* region, our results indicate that the relation between PUFA biosynthesis and CVDs is driven by changes in *FADS1* (not *FADS2*) expression among Europeans. In East Asians, the same *FADS1/2* variant was related to similar pleiotropic effects on the phenome (e.g. lipid, glycemic and blood cell traits) compared with Europeans, although the relation with CVDs was unclear as most effect estimates were either imprecisely estimated (e.g. AF) or, for CAD, in the opposite direction in East Asians compared with results in Europeans.

By triangulating multiple approaches, our results are compatible with higher LDL cholesterol (and possibly glucose) being a downstream effect of higher D5D activity (coded by *FADS1*) instead of being explained by confounding by LD or by the co-expression of other genes in the region. Given the well-established role of *FADS1*/D5D activity in PUFA biosynthesis and the well-known involvement of LDL cholesterol in the etiology of multiple CVDs, this strengthens the evidence for a causal relationship and provides a putative mediating pathway for the effect of PUFA biosynthesis on the risk of CVDs.

### Previous literature

The relation between fatty acids and CVDs has been explored in classical observational studies, randomized controlled trials and Mendelian randomization studies. Most previous studies have focused on CAD and, to a lesser extent, on stroke; therefore, other types of CVD endpoints, such as HF and AF, remained under explored.

Previous meta-analyses of classical observational studies indicate that higher circulating long-chain omega-3 and omega-6 PUFA are either not associated or are associated with lower risk of CAD and stroke (39–43), whereas higher circulating MUFA and SFA are either not associated or are associated with higher risk of CAD and stroke (39,40). Recent systematic reviews of randomized controlled trials of dietary advice or supplementation of omega-3 and omega-6 PUFA have suggested little to no benefit in reducing the risk of CVDs (44–46). However, most studies included in these systematic reviews were at moderate to high risk of bias and there is large uncertainty on the evidence linking PUFA to some cardiovascular outcomes (44–46). It is important to emphasize that comparing our findings to previous classical observational and randomized controlled trials deserves caution as our genetic instruments have a broad impact on the fatty acids pool and, therefore, cannot be used to make inferences about individual fatty acids/fatty acids classes. As an example, higher D5D activity (instrumented by rs174546) is related to higher longer chain omega-3 and omega-6 PUFA (e.g. AA, EPA and DHA) but lower shorter chain omega-3 and omega-6 PUFA (e.g. LA and ALA).

Several GWAS have reported that SNPs within the *FADS1/2* locus are associated with cardiovascular risk factors (e.g. LDL cholesterol and triglycerides) (19,47,48), and previous Mendelian randomization studies have reported that longer and shorter chain PUFA are related to risk of CVDs in contrasting directions among Europeans, including CAD in CARDIoGRAMplusC4D and UK Biobank (26,27,49), ischemic stroke in MEGASTROKE and UK Biobank (28,29) and VT in UK Biobank (29). Our findings expand on previous Mendelian randomization studies by implicating higher D5D activity in the development of a wide range of CVDs among Europeans in the largest available samples to date (up to 1 153 768 individuals). In addition, to our knowledge, this is the first Mendelian randomization study to report a potential protective effect of higher SCD activity on CAD among Europeans and to comprehensively explore the relation between D6D activity (coded by *FADS2*) and CVDs among East Asians.

### Plausibility of Mendelian randomization assumptions

A major challenge in Mendelian randomization studies is the unprovable assumption that the estimated effect of the genetic instrument on the outcome is mediated by the exposure and that it is not biased by horizontal pleiotropy, population stratification, assortative mating, indirect genetic effects or selection bias (38,50,51). We assessed the plausibility that our findings were explained by these sources of bias through a series of sensitivity analyses.

To mitigate bias owing to horizontal pleiotropy (i.e. the genetic instrument influences exposure and outcome via independent pathways), we have restricted our analyses to genetic variants near genes with well-established role in fatty acids biosynthesis. We have confirmed that these variants have the expected impact on the circulating fatty acids pool and on the expression of the target genes in key tissues (except for *FADS1* in whole blood). Previous evidence confirms that the selected *FADS1/2* and *SCD* variants, or variants in high LD, are related to changes in fatty acids composition across multiple sites, including adipose tissue (52–54), brain (55) and liver (56). Genetic colocalization and MVMR (adjusting for co-expressed genes in the region) supported a causal relation between D5D activity, VT and LDL cholesterol and between SCD activity and CAD. It is important to note that we were likely underpowered to test colocalization between fatty acids biosynthesis and CVD outcomes. Where there was evidence that the selected genetic variant was associated with the expression of non-target genes in the region in a given tissue, findings from MVMR were consistent with expression of the target gene (i.e. *FADS1*) having direct effects on the outcome.

Confounding could be introduced in Mendelian randomization studies owing to population stratification, assortative mating and indirect genetic effects. Of these factors, population stratification is likely to be of the most concern for this study (51). Despite attempts to control for population structure in genetic association data, there could still be residual population structure (36). We showed that within-sibship associations of *FADS1/2*, *ELOVL2* and *SCD* variants with established cardiovascular risk factors were broadly similar to estimates from unrelated individuals, suggesting that our results are unlikely to be affected by population stratification, assortative mating or indirect genetic effects of parents.

Non-random sample selection may introduce bias in Mendelian randomization studies, especially if the mechanism of selection depends on the exposure and/or outcome (38,57). Using a positive control approach, we were able to identify the expected effect of well-established risk factors on CVDs across studies contributing with data on CVD endpoints, which is reassuring given our concerns that case–control ascertainment could introduce bias in the analyses. Although results from the positive control approach argue against selection being a major source of bias in this study, we cannot fully rule out that selection might have introduced some bias in our analyses as bias owing to selection will depend on context-specific causal structures underlying the data under consideration.

### Implications

Our findings are supportive of the involvement of fatty acids biosynthesis, especially D5D and SCD activity, in the etiology of CVDs. Further work is needed to understand the precise underlying mechanism(s).

The relation between D5D activity and CVDs is plausibly mediated by one or more fatty acids involved in the PUFA biosynthesis pathway. Given the ubiquitous impact of higher D5D activity on the circulating PUFA pool, we cannot pinpoint which specific fatty acids are driving these effects. For illustration, higher D5D activity decreases LA and ALA (and other omega-3 and omega-6 PUFA upstream of the reaction catalyzed by D5D). Lower LA may relate to unfavorable metabolic changes, such as higher plasma LDL cholesterol, apolipoprotein B, and triglycerides and hemoglobin A1c (2,58) and, therefore, is a plausible mediator of the relation between higher D5D activity and higher CVDs risk. On the other hand, higher D5D activity increases long-chain PUFA, such as AA, which influences key membrane/tissue functions, such as membrane fluidity, the activity of membrane-bound receptors, transport proteins and signal transmission (59), and is a precursor for eicosanoids (e.g. prostaglandins, leukotrienes and thromboxane), which are involved in inflammation, platelet aggregation and vascular remodeling (60).

The putative mechanisms underpinning the relation SCD activity and CAD in humans are unclear. *Scd-1* deficient rodents are protected against diet-induced obesity, insulin resistance and hepatic steatosis (61–63) but show increased inflammation and atherogenesis (63,64). The putative protective effect of higher SCD activity on CAD might be related to lower availability of PA and consequent lower production of its toxic metabolites, such as ceramides (65).

### Conclusions

We found supportive evidence for an involvement of PUFA and MUFA biosynthesis in the etiology of CVDs. Our study illustrates the power of integrating multiple approaches to improve causal inference on the role of modifiable risk factors in the development of CVDs.

## Materials and Methods

### Data sources

The study included data from multiple consortia of genetic association studies (66–70) and biobanks (71–77).

#### Genetic associations with CVDs

The outcomes of interest were (prevalent/incident) CAD, ischemic stroke, hemorrhagic stroke, HF, AF, PAD, AA, VT and AVS.

Summary data for the association between genetic variants and these CVD endpoints was obtained from UK Biobank, FinnGen (release 4), BioBank Japan and several large-scale GWAS of CVD outcomes. If genetic association data on a cardiovascular endpoint were available from two or more independent datasets of individuals from the same genetic ancestry (i.e. UK Biobank and FinnGen), genetic association estimates were pooled across data sources using fixed-effect meta-analysis with inverse variance weights. Characteristics of studies and criteria for case definition are detailed in Supplementary Material, Table S8 and Supplementary Methods.

For individuals of European ancestry only/predominantly (i.e. UK Biobank, FinnGen and large-scale genetic association consortia), data were available on all outcomes of interest: CAD (*N* cases/controls = 123 668/702156), ischemic stroke (*N* cases/controls = 53 395/1030253), hemorrhagic stroke (*N* cases/controls = 4558/627188), HF (*N* cases/controls = 64 696/1089072), AF (*N* cases/controls = 77 945/1067430), PAD (*N* cases/controls = 9836/627950), AA (*N* cases/controls = 9735/730073), VT (*N* cases/controls = 25 284/616235) and AVS (*N* cases/controls = 2844/461776).

For individuals of East Asian ancestry (i.e. BioBank Japan), cardiovascular outcomes data were available for CAD (*N* cases/controls = 29 319/183 134), ischemic stroke (*N* cases/controls = 17 671/192 383), hemorrhagic stroke (*N* cases/controls = 2820/192 383), HF (*N* cases/controls = 9413/203 040), AF (*N* cases/controls = 8180/28 612) and PAD (*N* cases/controls = 3593/208 860).

#### Genetic associations with CVD risk factors

Other outcomes of interest were eight well-established risk factors for CVDs (i.e. LDL cholesterol, triglycerides, systolic, diastolic blood pressure, fasting glucose, type 2 diabetes, smoking and body mass index). Genetic association data for these risk factors were extracted for Europeans from a large-scale GWAS for type 2 diabetes (78) and UK Biobank for the other risk factors and for East Asians from BioBank Japan using the IEU OpenGWAS project database (79).

#### Genetic associations with circulating fatty acid concentration

For European ancestry individuals, we used genetic association data on circulating fatty acids from The Cohorts for Heart and Aging Research in Genomic Epidemiology (CHARGE) consortium, which has high resolution profiling of circulating fatty acids (*N* = 26 fatty acids measures) measured in 8631–8866 individuals (15–17). We also used data from two other genetic association meta-analyses on circulating fatty acids (18,19) for assessing replication as detailed in Assessing the impact of genetic instruments on the fatty acids pool’.

For East Asian ancestry individuals, we used genetic association data on fatty acids from the Singapore Chinese Health Study (SCHS) for circulating PUFA (*N* = 1361) (21) and from a metanalysis of the Nutrition and Health of Aging Population in China and the Chinese ancestry individuals of the Multi-Ethnic Study of Atherosclerosis for RBC or circulating SFA and MUFA (*N* = 3521) (80,81).

Characteristics of these studies are detailed in Supplementary Material, Table S9.

### Data analysis

#### Selection of genetic instruments indexing fatty acids biosynthesis

We selected genetic variants mapping to genes that have well-characterized roles in fatty acids biosynthesis and have been previously reported by GWAS to influence circulating fatty acids (Fig. 1). In Europeans, three genomic regions were eligible, harboring *FADS1/2*, *ELOVL2* and *SCD* genes, whereas, in East Asians, only the *FADS1/2* locus was strongly associated with circulating fatty acids, which may be related to the modest sample size available for East Asians (*N* = 1361–3521). *FADS1/2* were considered as one single genomic region since these genes are in close proximity to each other (i.e. 0.8 kb) on the long arm of human chromosome 11.

Genetic variants regulating the expression/activity of *FADS1/2*, *ELOVL2* and *SCD* will affect multiple fatty acids on the same pathway and, in some cases, on different pathways with reactions catalyzed by the same enzymes (Fig. 1). As a result, selecting genetic variants for individual fatty acids can be highly redundant. Instead, we selected the genetic variant (±500 kB of the target gene) most strongly related (*P*-value < $5\times{10}^{-8}$) to a proxy of the enzyme activity (i.e. the ratio between fatty acids that are the product and the substrate of a reaction catalyzed by a particular enzyme) within each genomic locus (Table 3). As an example, a higher ratio of AA to DGLA would indicate more active conversion owing to higher expression/activity of D5D, the enzyme coded by *FADS1*.

**Table 3:** Genomic region, target gene and corresponding proxy of enzyme activity

Locus	Chr	Enzyme	Enzyme activity proxy^a^	Fatty acids class	Ancestry
*FADS1*	11	D5D	AA:DGLA	PUFA n-6	Europeans
*FADS2*	11	D6D	GLA:LA DGLA:LA	PUFA n-6	Europeans East Asians
*ELOVL2*	6	ELOVL2	DHA:DPAn3	PUFA n-3	Europeans
*SCD*	10	SCD	POA:PA	MUFA/SFA	Europeans

^a^Enzyme activity was proxied based on enzyme-specific product to substrate ratio using data from circulating fatty acids. AA: arachidonic acid; DGLA: dihomo-γ-linolenic acid; DHA: docosahexaenoic acid; DPA: docosapentaenoic acid; *ELOVL2:* elongase 2; *FADS:* fatty acids desaturase; GLA: γ-linoleic acid; LA: linoleic acid; MUFA: monounsaturated fatty acids; PA: palmitic acid; POA: palmitoleic acid; PUFA: polyunsaturated fatty acids; SFA: saturated fatty acids; *SCD:* stearoyl-CoA desaturase.

For European ancestry individuals, we derived genetic association data for proxies of enzyme activity by applying the ‘Genome-wide Inferred Study’ (GWIS) method to genetic association data for circulating fatty acids from the CHARGE consortium (15–17) for the ratios of AA to DGLA (proxy of D5D activity), GLA to LA (proxy of D6D activity), DHA to DPA n-3 (proxy of ELOVL2 activity) and POA to PA (proxy of SCD activity). Briefly, GWIS approximates genetic association estimates for a new variable as a linear function of the allele frequencies, population means of measured traits (assumed to approximate the intercepts of the model) and genetic association estimates of measured traits. Corresponding standard errors can be derived using the Delta method having obtained the covariance matrix for effect estimates (82).

For East Asian ancestry individuals, the original GWAS investigators derived genetic association data for the proxies of enzyme activity from individual level data on circulating fatty acids (21), as follows: AA to DGLA (proxy of D5D activity) and DGLA to LA (proxy of D6D activity).

If the selected genetic variant was a palindromic SNP, it was replaced by a non-palindromic proxy variant in strong LD to avoid data harmonization problems in subsequent analyses. All SNP-trait associations were harmonized so that the allele associated with increasing enzyme activity was the effect allele, indicating more active conversion.

We approximated the *R*^2^, a measure of the variance in exposure explained by the genetic variant, and the *F* statistics, a measure of instrument strength (83), as follows:

$$ {R}^2=2\ast{\beta}_{gx}^2\ast \mathrm{MAF}\ast \left(1-\mathrm{MAF}\right), $$

$$ F=\left(\frac{n-k-1}{k}\right)\ast \left(\frac{R^2}{1-{R}^2}\right), $$

where ${\beta}_{gx}$ is the SNP-fatty acid trait association estimate (in SD units), MAF is the minor allele frequency, *n* is the sample size and *k* is the number of SNPs.

#### Assessing the impact of genetic instruments on the fatty acids pool

We assessed the impact of the selected genetic instruments on the circulating fatty acids pool in the discovery samples (i.e. CHARGE in Europeans and SCHS in East Asians) for internal validation. Among European ancestry individuals, we could test for replication in two independent datasets (external validation) (18,19).

#### Mendelian randomization analysis

For each cardiovascular outcome, we used the Wald ratio method (84,85) to estimate the odds ratio of disease for each standard unit increase in the proxy of enzyme activity by dividing estimates for the genetic association with cardiovascular outcome by estimates for the genetic association with enzyme activity as follows:

$$ {\beta}_{MR}=\frac{\beta_y}{\beta_x} $$

and corresponding standard error:

$$ {\mathrm{SE}}_{MR}=\frac{{\mathrm{SE}}_y}{\beta_x}, $$

where ${\beta}_y$ and ${\beta}_x$ are the coefficients for the association of the genetic variant with the outcome (*Y*) and the exposure (*X*), respectively, and ${\mathrm{SE}}_y$ is the standard error for the association of the genetic variant with *Y*.

We used a Bonferroni correction to account for the maximum number of outcomes available (*P*-value = 0.05/9 outcomes = 0.00556 in Europeans). The same approach was used to estimate the relation between enzyme activity and the eight well-established risk factors for CVDs using Bonferroni correction to account for multiple testing (*P*-value = 0.05/8 risk factors = 0.00625). We use these *P*-value thresholds simply as a heuristic for highlighting associations worthy of follow-up. Mendelian randomization analyses were performed using R software version 3.6.2 (R Foundation for Statistical Computing) including the TwoSampleMR R package (86).

#### Genetic colocalization

We used coloc (87), a method for pairwise genetic colocalization analysis, to test whether the same genetic variant influences fatty acids biosynthesis and CVDs risk or risk factor. Coloc enumerates all possible configurations of causal variants for each of two traits (e.g. fatty acid- and CVD-related traits) and uses a Bayesian approach to calculate support for each causal model (H_1_: association with trait 1 only; H_2_: association with trait 2 only; H_3_: association with both traits owing to distinct causal variants; H_4_: association with both traits owing to a single shared causal variant; H_5_: no association). We restricted coloc analysis to a genomic region within a 500 kb window around each target gene (*FADS1/2*, *ELOVL2* and *SCD*) and assumed prior probabilities that any random SNP in the region is associated with trait 1 (p1 = $1\times{10}^{-4}$), trait 2 (p2 = $1\times{10}^{-4}$) or both traits (p12 = $1\times{10}^{-6}$). A PPA ≥ 70% for association with both traits owing to a single causal variant was considered as strong evidence for a shared genetic variant.

Coloc assumes a single causal variant in the genomic region, and, as a result, the presence of multiple conditionally independent SNPs within a region can affect the performance of the method. Therefore, where Coloc provided some evidence of distinct genetic signals (i.e. PPA for distinct genetic variants >30%), we also performed approximate conditional analyses using GCTA (88,89) (adjusting for the top SNP in the outcome dataset in each genomic region) and re-ran Coloc using the adjusted association estimates as a sensitivity analyses. Colocalization analyses were restricted to European datasets as the method assumes that samples are drawn from independent populations of similar genetic background (i.e. allele frequencies and LD pattern are identical), which was not the case for East Asians in our analyses since fatty acids and CVD data were derived from Singaporean Chinese and Japanese individuals, respectively.

#### Phenome-wide scan

We explored the potential mechanisms that might link the selected genetic variants to CVDs by using an automated phenome-wide scan tool from the IEU OpenGWAS project database (79) to test the association of the selected genetic variants with 32 534–34 465 (non-unique) traits for European ancestry individuals and 110 traits for Japanese individuals from BioBank Japan. We used a Bonferroni correction to account for multiple testing considering the maximum number of traits included in the phenome-wide scan in Europeans (*P*-value = 0.05/34465 = $1.5\times{10}^{-6}$) and East Asians (*P*-value = 0.05/110 = $4.5\times{10}^{-4}$).

#### Gene expression and tissue-specific analyses

We explored the influence of higher expression of the target genes (i.e. *FADS1*, *ELOVL2* and *SCD*), and their tissue specificity, on CVDs risk and risk factors in individuals of European ancestry by integrating expression quantitative trait loci (eQTL) data from Genotype-Tissue Expression (GTEx) version 8 with genetic association data for cardiovascular traits. We extracted eQTL data from the GTEx for multiple tissues of relevance to CVDs—i.e. subcutaneous/visceral adipose tissues, aorta/coronary/tibial arteries, heart, liver, pancreas and whole blood (*N* = 208–670 individuals per tissue) (90).

In step i, we performed a cross-tissue assessment of the association of the selected genetic variants with transcription of any genes in the region (aka *cis*-genes), defined as genes for which the transcription start site was 1 Mb away from the genetic variant. The *cis*-genes were selected for follow-up analysis if the *P*-value for the SNP-gene expression association was <5% false discovery rate threshold for each tissue.

In the step ii, we used coloc to test whether the same genetic variant influences fatty acids biosynthesis and expression of the target gene across tissues using the same approach described in ‘Genetic colocalization’.

In the step iii, we used MVMR to jointly model the expression of a target gene (i.e. *FADS1/2*, *ELOVL2*, and *SCD*) and a co-expressed *cis*-gene (identified as described in ‘step i’) on cardiovascular outcomes across tissues. This analysis allowed us to estimate the direct contribution of changes in the expression of each target gene where the selected genetic variant was related to co-expression of a non-target *cis*-gene. We selected independent eQTLs (*P* < $5\times{10}^{-5}$; *R*^2^ < 0.05; 1000 Genomes EUR reference population) for each combination of target gene (i.e. *FADS1/2*, *ELOVL2* and *SCD*) and non-target *cis*-gene and performed MVMR using the MVMR R package (91). MVMR models were estimated for each combination of target gene, co-expressed gene, outcome and tissue if (i) the target gene SNP (i.e. rs174546, rs2236212, and rs603424) was related to expression of non-target genes in that tissue (step i), (ii) more than two independent SNPs were selected for the analyses and (iii) the conditional *F* statistics for the target gene expression was equal or higher than 5.

#### Negative control outcomes

We tested the association between the selected genetic variants with two negative control outcomes, i.e. skin color and ease of skin tanning, using UK Biobank genetic association data deposited in the IEU Open GWAS Project (79). Since these traits could not conceivably be affected by fatty acids biosynthesis, any evidence for an association between genetic variants and these negative control outcomes would be indicative of residual population stratification (92).

#### Within-sibship analyses

We used data from a recent within-sibship GWAS, including up to 178 076 individuals (77 832 sibling pairs) from 23 cohorts, to evaluate if our findings are sensitive to population stratification, assortative mating and indirect genetic effects of parents. Within-family designs, such as parent-offspring trio or within-sibship models, control for variation in parental genotypes and so, are not affected by these potential biases (51,93,94).

We compared the within-sibship association of the selected genetic variants with cardiovascular risk factors (LDL cholesterol, triglycerides, systolic blood pressure, glycated hemoglobin, smoking and body mass index) with estimates from standard GWAS models in unrelated individuals (sample size ranging from 50 361 for glycated hemoglobin to 155 457 for body mass index). Data on CVD endpoints and other risk factors (i.e. diastolic blood pressure, fasting glucose and type 2 diabetes) were not available.

#### Positive control exposures

Several processes of sample selection, occurring from study design to data analyses, can result in selected samples not being representative of their target populations, which may bias causal inference, including when using Mendelian randomization (38). We were particularly concerned about selection owing to ascertainment of CVD status. As an example, BioBank Japan is a hospital-based study in which cases for CVDs, except AF, were compared with a control group, including a mixture of hospital-based (i.e. individuals diagnosed at health centers with other diseases) and community-based (i.e. individuals from population-based cohorts) controls as previously described (74). In addition, UK Biobank has a response rate of 5.5% and its participants have fewer self-reported health conditions and are more likely to be older, female, wealthier, leaner, non-smokers and non-drinkers than the general UK population (95).

To explore whether these processes of sample selection could bias our findings, we adopted a positive exposure control approach in which we used Mendelian randomization to estimate the effect of well-established cardiovascular risk factors (i.e. LDL cholesterol, triglycerides, systolic, diastolic blood pressure, glucose, type 2 diabetes, smoking and body mass index) on the risk of CVDs. If the effects estimated in the positive control analyses were compatible with what expected and were comparable across data sources, such analyses would argue against selection being a major source of bias.

## Supplementary Material

BorgesMC_MR_FAvsCVD_2022_05_16_SUPPLFIG_ddac153

BorgesMC_MR_FAvsCVD_2022_05_16_SUPPLMETHODS_ddac153

BorgesMC_MR_FAvsCVD_2022_05_16_SUPPLTABLES_ddac153

## Data access statement

Full genetic association data from GWAS of circulating fatty acids and of cardiovascular diseases can be accessed as described in Supplementary tables 8 and 9. Researchers can apply for access to the UK Biobank data via the Access management System (AMS) (https://www. ukbiobank.ac.uk/enable-your-research/apply-for-access).
